# Differences between medical student and faculty perceptions of the competencies needed for the first year of residency

**DOI:** 10.1186/s12909-017-1036-7

**Published:** 2017-11-09

**Authors:** Sophie Fürstenberg, Sigrid Harendza

**Affiliations:** 1 Department of Internal Medicine, University Medical Center Hamburg-Eppendorf, Hamburg, Germany; 2 Universitätsklinikum Hamburg-Eppendorf, III. Medizinische Klinik, Martinistr. 52, D-20246 Hamburg, Germany

**Keywords:** Competencies, Physicians, Postgraduate medical education, Residency, Undergraduate medical students

## Abstract

**Background:**

Different guidelines and frameworks like the CanMEDs model or entrustable professional activities (EPAs) describe competencies required for successful and professional work of residents. Not all competencies are of equal importance for graduates when they start their residency. The aim of this study was to evaluate the relevance of different competencies for a first year resident from the perspective of physicians and medical students.

**Methods:**

In an online study, 178 of 475 surgeons and internists including residents and attendings and 102 of 728 first and last year undergraduate medical students from the University Medical Center Hamburg-Eppendorf ranked 25 competencies according to their relevance for entrustment decisions in first year residents. The rankings of the competencies by residents and attendings and by first year and last year medical student were compared. Additionally, the rankings were also compared to the literature.

**Results:**

Physicians and medical students rated ‘Responsibility’ as the most important competency for first year residents. Physicians ranked ‘Teamwork and collegiality’ and ‘Structure, work planning and priorities’ within the top 10 competencies significantly higher than medical students. The competency ranks between attendings and residents only showed one significant difference between attendings and residents, where ‘Coping with mistakes’, was ranked significantly higher by residents. Medical students ranked ‘Active listening to patients’, ‘Advising patients’ and ‘Handling emotions of patients and their relatives’ significantly higher than physicians. Final year students ranked ‘Structure, work planning and priorities’, ‘Coping with mistakes’, and ‘Verbal communication with colleagues and supervisors’ significantly higher than first year students.

**Conclusions:**

Even though physicians and medical students agree that ‘Responsibility’ is the most important competency for entrustment decisions in the first year of residency, medical students rate competencies regarding patient communication very highly while physicians rate competencies required for patient managements significantly higher for entrustment decision. Undergraduate medical curricula seem to prepare students well with respect to patient-centeredness but need to be developed more specifically to prepare students equally well for patient management competencies which are required in the first year of residency for entrustment decisions from the attendings perspective.

## Background

The CanMEDS model of postgraduate education includes seven specific roles of a physician based on competencies, which will enable the trainees to fulfil the respective roles in their daily work [1]. Based on the CanMEDS outline, competency-based postgraduate trainings have been developed in different countries and for different specialties, e.g. for internal medicine and surgery [2, 3]. Many medical schools are also in the process of reshaping their undergraduate medical curriculum with a focus on competency-based learning and entrustable professional activities (EPA) to improve graduates’ preparedness for practice [4–8]. Each EPA is based on a defined number of competencies [9]. When supervisors of postgraduate training programs were asked to rate the preparedness for work of their medical graduates, they evaluated students who graduated from a vertically integrated curriculum higher with respect to the capability to work independently, to solve medical problems, to manage unfamiliar medical situation, to prioritize tasks, and to estimate when they need to consult their supervisors [10]. When Dutch medical students from a vertically integrated undergraduate medical curriculum were asked in their final year to rate the importance of competencies related to the seven CanMEDS roles, professionalism and communication received the highest scores [11].

In daily work, supervisors trust their trainees with specific tasks and decide when supervision is needed [12, 13]. However, Raymond et al. found that new residents performed a wide range from routine to more complex tasks unsupervised [14]. In a study by Touchie et al. first year residents reported that they performed certain EPAs with less supervision than their clinical supervisors expected [15]. Usually, specific tasks require specific skills and general features, which are independent of the specific task, the competencies or facets of competence [16]. A Delphi study with two rounds based on the opinion of experienced physician educators from the Netherlands identified 25 competencies that are important for clinical entrustment decisions [16]. When Dutch and German clinicians who also held an academic chair in medical education and supervised residents were asked to rank the 25 competencies with a specific scoring system, they reached almost full agreement on the top 10 competencies [17]. These included ‘Scientific and empirical grounded method of working’, ‘Knowing and maintaining own personal bounds and possibilities’, ‘Active professional development’, ‘Teamwork and collegiality’, ‘Active listening to patients’, and ‘Verbal communication with colleagues and supervisors’ [17]. However, the number of participants with eight in each group was small and it is still unknown, how physicians or medical students would rank these competencies and whether they have different opinions in their evaluation of the competencies with respect to their importance for first year residents. We hypothesize that physicians and medicals students in the different stages of their training differ in their assessment of specific competencies relevant for entrustment decisions in the first year of residency. The aim of our study was to assess whether such a difference can be identified to provide curricula planners with the range of perspectives on competencies needed for the first year of residence and to extract the most important competencies needed in entrustable professional activities for first year residents.

## Methods

To compare the perspective on the importance of different competencies for entrustment decisions in first year residents, physicians and undergraduate medical students from the University Medical Center Hamburg-Eppendorf (UKE) were invited to participate in this study. The UKE employ approximately 2400 physicians and teaches approximately 2300 undergraduate medical students. In total, 475 physicians (all 234 residents and all 241 attendings) from the following surgical and internal medicine departments with six-year residency programs were approached: Cardiovascular Surgery; General, Visceral and Thoracic Surgery; Hepatobiliary Surgery and Transplantation; Neurosurgery; Oral and Maxillofacial Surgery; Plastic, Reconstructive and Aesthetic Surgery; Trauma, Hand and Reconstructive Surgery; Vascular Surgery; Cardiology; Endocrinology, Gastroenterology; Hematology, Nephrology, Oncology, Pulmonology, and Rheumatology. Furthermore, 728 undergraduate medical students (360 in their first year, 368 in their final year) received an invitation to participate. All subjects received the invitation with a link to the questionnaire by email and participation was anonymous and voluntary.

The online questionnaire included one page with sociodemographic questions: age, gender, and position (resident or attending) for physicians and age, gender, current semester, and month of final year for students. On a second page, the 25 competencies needed for entrustment decisions in first year residents, which were established and ranked previously [17] were placed in random order and further instructions were given. Participants were asked to drag and drop every competency from the left to the right side of the computer screen in a way that eventually five competencies were placed in five categories: “most important” (5 points), “very important” (4 points), “important” (3 points), “less important” (2 points), “least important” (1 point). A full description of every competency [16] was provided in an additional window for reference. When all competencies were placed in a category the questionnaire could be sent back. The survey took place in the fall of 2016 and lasted for six weeks. A reminder was sent by email after the second and the fourth week. Means and standard deviations were calculated for the different groups of participants. Statistical analysis included t-tests and a one-way analysis of variance (ANOVA) to study differences between the subject-groups.

## Results

In total, 1203 physicians and students had been invited to participate in this study. 293 started and 178 participants completed the questionnaire (response rate 14.8%), 76 physicians (response rate 15.9%) – 56 internists and 20 surgeons – and 102 undergraduate medical students (response rate 14.0%). Table 1 shows the gender distribution of all participants. Among the 76 physicians were 36 attendings (age: 43.3 ± 10.3 years) and 40 residents (age: 31.8 ± 3.3 years). Of the 102 undergraduate medical students 55 were in their first year (age: 21.6 ± 4.8 years) and 47 were in their final year (age: 28.2 ± 4.8 years).

**Table 1:** Gender distribution of the participating physicians and medical students

Gender	Physicians				Medical students			
	Attendings (*N* = 40)		Residents (*N* = 36)		Final year (*N* = 47)		First year (*N* = 55)	
	n	%	n	%	n	%	n	%
Male	24	60.0	21	58.3	21	44.7	17	30.9
Female	16	40.0	13	36.1	26	55.3	38	69.1
Not specified			2	5.6				

Table 2 shows the ranking of the competencies relevant for entrustment decisions in first year residents, comparing the votes of all participating physicians with all participating students. Ranks 1 to 10 are marked in bold and significant differences in rank positions are marked, indicating the higher rank. Physicians and students identified the same eight of the top 10 competencies with ‘Reliability’ being the most important competence in both groups. Within the top 10 competencies physicians ranked ‘Teamwork and collegiality’ and ‘Structure, work planning and priorities’ significantly higher than medical students and medical students ranked ‘Active listening to patients’ significantly higher (rank 3) compared with physicians (rank 8). Students ranked ‘Advising patients’ and ‘Handling emotions of patients and their relatives’ with significantly higher scores among the top 10 while these competencies do not appear within the top 10 of physicians. ‘Scientifically and empirically grounded method of working’, which appears with a significantly higher rating on rank 6 in the physicians’ list marks rank 18 in the students’ list. Furthermore, students ranked ‘Attention to individual patient background’, ‘Respecting privacy and autonomy of the patient’, ‘Attention to psychosocial aspects of health problems’, and ‘Active health promotion’ significantly higher than physicians while physicians rated ‘Written (and digital) account/report to colleagues and supervisors’ significantly higher than students.

**Table 2:** Ranking order of the 25 competencies by all participating physicians and medical students

Competency	Physicians (*N* = 76)		Medical students (*N* = 102)	
	rank	M ± SD	rank	M ± SD
Responsibility	**1**	4.47 ± 0.77	**1**	4.25 ± 1.08
Teamwork and collegiality	**2**	4.12 ± 1.01*	**5**	3.74 ± 1.19
Structure, work planning and priorities	**3**	4.08 ± 1.30**	**7**	3.47 ± 1.43
Knowing and maintaining own personal bounds and possibilities	**4**	4.07 ± 1.26	**4**	3.84 ± 1.33
Empathy and openness	**5**	4.03 ± 0.99	**2**	4.24 ± 1.10
Scientifically and empirically grounded method of working	**6**	3.62 ± 1.46***	18	2.71 ± 1.52
Coping with mistakes	**7**	3.62 ± 1.05	**6**	3.61 ± 1.06
Active listening to patients	**8**	3.36 ± 1.12	**3**	3.99 ± 1.09***
Verbal communication with colleagues and supervisors	**9**	3.30 ± 1.35	13	3.04 ± 1.27
Ethical awareness	**10**	3.29 ± 1.37	**9**	3.20 ± 1.44
Active professional development	11	3.11 ± 1.39	15	2.82 ± 1.28
Coping with uncertainty	12	3.03 ± 1.33	17	2.75 ± 1.33
Advising patients	13	3.01 ± 1.19	**8**	3.37 ± 1.18*
Safety and risk management	14	3.01 ± 1.31	19	2.65 ± 1.37
Attention to individual patient background	15	2.70 ± 1.15	11	3.12 ± 1.28*
Written (and digital) account/report to colleagues and supervisors	16	2.68 ± 1.25***	22	2.08 ± 1.11
Adapted informing of patients	17	2.67 ± 1.12	14	2.96 ± 1.33
Handling emotions of patients and their relatives	18	2.66 ± 1.10	**10**	3.17 ± 1.14**
Respecting privacy and autonomy of the patient	19	2.59 ± 1.28	12	3.11 ± 1.21**
Continuity in the care process	20	2.24 ± 1.26	23	2.01 ± 1.09
Attention to relatives and caregivers	21	2.17 ± 0.87	21	2.25 ± 1.17
Role differentiation	22	2.04 ± 1.26	24	1.84 ± 1.24
Attention to psychosocial aspects of health problems	23	1.83 ± 1.02	16	2.76 ± 1.04***
Active health promotion	24	1.79 ± 1.11	20	2.45 ± 1.33***
Financial and social awareness	25	1.58 ± 0.93	25	1.60 ± 0.95

*: *p* < .05, **: *p* < .01, ***:*p* < .001; ranks 1–10 are marked in bold

The competency ranks compared between attendings and residents (Table 3) only showed one significant difference between attendings and residents, i.e. ‘Coping with mistakes’, which was ranked significantly higher by residents. ‘Verbal communication with colleagues and supervisors’ as well as ‘Safety and risk management’ were ranked among the top 10 by residents but not by attendings. However, no significant difference was found for these two competencies between residents and attendings.

**Table 3:** Ranking order of the 25 competencies by attendings and residents

Competency	Attendings (*N* = 40)		Residents (*N* = 36)	
	rank	M ± SD	rank	M ± SD
Responsibility	**1**	4.63 ± 0.67	**1**	4.31 ± 0.86
Teamwork and collegiality	**2**	4.13 ± 1.02	**3**	4.11 ± 1.01
Knowing and maintaining own personal bounds and possibilities	**3**	4.13 ± 1.14	**4**	4.00 ± 1.39
Empathy and openness	**4**	4.10 ± 1.06	**5**	3.94 ± 0.92
Structure, work planning and priorities	**5**	4.00 ± 1.34	**2**	4.17 ± 1.28
Scientifically and empirically grounded method of working	**6**	3.78 ± 1.42	**8**	3.44 ± 1.50
Active listening to patients	**7**	3.45 ± 1.15	11	3.25 ± 1.08
Coping with mistakes	**8**	3.38 ± 1.08	**6**	3.89 ± 0.95*
Advising patients	**9**	3.25 ± 1.13	15	2.75 ± 1.23
Ethical awareness	**10**	3.25 ± 1.37	**9**	3.33 ± 1.39
Active professional development	11	3.23 ± 1.35	13	2.97 ± 1.44
Verbal communication with colleagues and supervisors	12	3.18 ± 1.32	**7**	3.44 ± 1.38
Coping with uncertainty	13	2.85 ± 1.31	12	3.22 ± 1.33
Written (and digital) account/report to colleagues and supervisors	14	2.83 ± 1.32	18	2.53 ± 1.16
Safety and risk management	15	2.75 ± 1.17	**10**	3.31 ± 1.41
Respecting privacy and autonomy of the patient	16	2.73 ± 1.18	19	2.44 ± 1.38
Attention to individual patient background	17	2.65 ± 1.17	16	2.75 ± 1.16
Handling emotions of patients and their relatives	18	2.63 ± 1.08	17	2.69 ± 1.14
Adapted informing of patients	19	2.58 ± 1.08	14	2.78 ± 1.17
Continuity in the care process	20	2.25 ± 1.35	21	2.22 ± 1.17
Attention to relatives and caregivers	21	2.05 ± 0.78	20	2.31 ± 0.95
Role differentiation	22	2.00 ± 1.28	22	2.08 ± 1.25
Attention to psychosocial aspects of health problems	23	1.98 ± 1.10	25	1.67 ± 0.93
Active health promotion	24	1.80 ± 1.26	23	1.78 ± 0.93
Financial and social awareness	25	1.45 ± 0.78	24	1.72 ± 1.06

*: *p* < .05; ranks 1–10 are marked in bold

Among the top 10 competencies, final year students ranked ‘Structure, work planning and priorities’ (rank 3), ‘Coping with mistakes’ (rank 4), and ‘Verbal communication with colleagues and supervisors’ (rank 8) significantly higher than first year students (rank 13, 8, and 16, respectively). First year students rated ‘Respecting privacy and autonomy of the patient’ and ‘Adapted informing of patients’ significantly higher among the top 10 compared with final year students. Other significant differences with higher ratings by final year students were ‘Coping with uncertainty’ and ‘Written (and digital) account/report to colleagues and supervisors’ while first year students ranked ‘Active health promotion’ significantly higher than final year students. Compared to final year students, residents ranked ‘Safety and risk management’ (rank 10) significantly higher than final year students (rank 19), while final year students ranked ‘Active listening to patients’ (rank 6 versus 11), ‘Handling emotions of patients and their relatives’ (rank 11 versus 17) and ‘Attention to psychosocial aspects of health problems’ (rank 18 versus 25) significantly higher than residents (Table 4).

**Table 4:** Ranking order of the 25 competencies by final year and first year medical students

Competency	Final year (*N* = 47)		First year (*N* = 55)	
	rank	M ± SD	rank	M ± SD
Empathy and openness	**1**	4.11 ± 1.13	**2**	4.35 ± 1.08
Responsibility	**2**	4.09 ± 1.19	**1**	4.38 ± 0.95
Knowing and maintaining own personal bounds and possibilities	**3**	4.04 ± 1.32	**4**	3.67 ± 1.33
Structure, work planning and priorities	**4**	3.96 ± 1.28**	13	3.05 ± 1.43
Coping with mistakes	**5**	3.91 ± 1.00**	**8**	3.35 ± 1.06
Active listening to patients	**6**	3.89 ± 1.07	**3**	4.07 ± 1.10
Teamwork and collegiality	**7**	3.87 ± 1.10	**5**	3.62 ± 1.25
Verbal communication with colleagues and supervisors	**8**	3.36 ± 1.15*	16	2.76 ± 1.32
Ethical awareness	**9**	3.32 ± 1.37	12	3.09 ± 1.51
Advising patients	**10**	3.28 ± 1.21	**6**	3.45 ± 1.15
Handling emotions of patients and their relatives	11	3.21 ± 1.16	11	3.13 ± 1.14
Coping with uncertainty	12	3.11 ± 1.48*	20	2.44 ± 1.10
Attention to individual patient background	13	3.02 ± 1.26	**10**	3.20 ± 1.31
Respecting privacy and autonomy of the patient	14	2.81 ± 1.19	**7**	3.36 ± 1.18*
Scientifically and empirically grounded method of working	15	2.79 ± 1.53	19	2.64 ± 1.52
Active professional development	16	2.70 ± 1.28	15	2.93 ± 1.27
Adapted informing of patients	17	2.60 ± 1.15	**9**	3.27 ± 1.41*
Attention to psychosocial aspects of health problems	18	2.57 ± 1.04	14	2.93 ± 1.02
Safety and risk management	19	2.57 ± 1.41	18	2.71 ± 1.34
Written (and digital) account/report to colleagues and supervisors	20	2.49 ± 1.14***	24	1.73 ± 0.97
Attention to relatives and caregivers	21	2.13 ± 1.10	21	2.35 ± 1.24
Active health promotion	22	2.11 ± 1.18	17	2.75 ± 1.39*
Continuity in the care process	23	1.89 ± 0.98	22	2.11 ± 1.18
Role differentiation	24	1.68 ± 1.14	23	1.98 ± 1.31
Financial and social awareness	25	1.49 ± 0.80	25	1.69 ± 1.05

*: *p* < .05, **: *p* < .01, ***:*p* < .001; ranks 1–10 are marked in bold

## Discussion

This study aimed to explore the perspective of physicians and medical students on the importance of different competencies for entrustment decisions in first year residents to give curricular planner some insight whether the perspectives of students and physicians resemble the realistic learning situation of first year residents and how the perspectives could be integrated in the undergraduate medical curriculum. Independently of the groups of participants, competencies like ‘Responsibility’, ‘Empathy and openness’, and ‘Knowing and maintaining own personal bounds and possibilities’ ranked very high among the overall top 10 competencies and comprise important aspects of professionalism [18]. Medical students and physicians might have chosen them as being important for the first year of residency because they resemble features of a physician’s professional behavior [19]. This could be taken as a hint that aspects of professionalism should be included in the undergraduate medical curriculum, preferably in a longitudinal way because more challenging professional situations will arise the further students progress in their education. In general, a similar set of top 10 competencies for the first year of residency was chosen by physician educators from the Netherlands and Germany in an earlier study [17] but some differences in contrast with the current ranking (e.g. ‘Responsibility’: rank 1 in this study, rank 10 in the earlier study) can be observed.

Medical students ranked competencies connected with direct patient care (e.g. ‘Active listening to patients’, ‘Handling emotions of patients and their relatives’) significantly higher than physicians. The choice of these patient-centered competencies might be due to students’ learning experiences in our undergraduate medical curriculum and their preparedness to practice patient communication, which is rated highly by students from different undergraduate medical curricula [20]. In a medical students’ appraisal of CanMEDS roles, the communicator role was rated highest and the manager role was rated lowest [11]. In our study, physicians ranked competencies related to patient management like ‘Structure, work planning and priorities’ and ‘Written (and digital) account/report to colleagues and supervisors’ significantly higher compared with medical students. From their perspective and experience as supervisors, they can judge that management skills are required from the very first day of residence when working in interprofessional teams and seem to be largely undeveloped in medical education [21]. Documentation is an important competency of patient management in residency but students feel least prepared for this competency independently of the type of their medical school training [20]. However, documentation can be improved in medical school with feedback [21] to prepare graduates better for this competency required in the first year of residency. Furthermore, medical students are well aware that certain disciplines, e.g. internal medicine, require more paperwork and charting than others [22] and appreciate training in such patient management competencies at medical school [23]. Additionally, patient management plays a great role in the core entrustable professional activities that have been defined for entering residency [24]. Hence, further development towards competence-based undergraduate medical curricula seems to be an important direction to prepare students for the task they will encounter in residency even though competence-based medical education has been criticized for being reductionistic [25].

Compared to first year students, final year students are significantly more aware that management competencies play an important role in the first year of residency. In this respect, they resemble the residents’ rating much more than the students’ rating. Our findings are underscored by a study, which acknowledged that final year medical students reported an increase in the management of procedural skills after completing a pre-internship course [26]. Additionally, final year students rated ‘Coping with mistakes’ and ‘Coping with uncertainty’ significantly higher for entrustment decisions in the first year of residency than first year students. ‘Coping with mistakes’ was also the only competency which was rated significantly higher by residents compared with attendings. On the other hand, first year medical students rate competencies related to patient care significantly higher than final year students. Their understanding of residents’ competencies is highly related to patient care, the center of their undergraduate studies being important for their identity formation as future physicians [27]. However, with experience, final year students and young residents learn that mistakes will occur and can occur easily, especially when salient clinical features distract a physician’s attention [28]. The competency to cope with mistakes is also important for clinical reasoning strategies where diagnostic errors can occur with analytical and non-analytical thinking [29]. First year medical students who struggle to acquire medical knowledge and skills cannot foresee how important coping with mistakes that will happen in clinical reasoning will be for entrustment decisions in a first year resident. A special training for residents can enhance their awareness for cognitive bias and reduce the number of cognitive errors [30], which ease the entrustment in a resident. Even though all 25 competencies will be necessary for a physician’s daily work, it might be worthwhile in curriculum planning to focus particularly on the top 10 competencies identified in this study during the final year of undergraduate medical education to ease the transition into residency.

### Strengths and weaknesses of this study

A strength of our study is that a larger cohort of physicians and medical students performed and confirmed the ranking of competencies for the first year of residency, which was based in a previous study only on the opinion of very few educational specialists. However, the overall response rate of approximately 15.5% in our study was still low, probably due to the time consuming task of reading and ranking the competency definitions properly. As far as physicians are concerned, we only included surgeons and internists because they resemble the largest number of physicians involved in the highest number of teaching hours, which is another limitation of our study. It could have led to a bias in the ranking that physicians from other specialties were not included. Furthermore, the number of female physicians in leading positions who participated in our study was higher than the percentage of women in leadership positions in the invited departments, which could also have led to a bias. Despite these limitations, it is a strength of our study that physicians and medical students were involved in the evaluation of the competencies required for entrustment decisions in first year residents, which provides useful insights for further development of undergraduate medical curricula.

## Conclusions

While medical students rate competencies regarding patient communication very highly for entrustment decisions in first year residents physicians rate competencies required for patient managements very highly. Apparently, our undergraduate medical curriculum prepares medical students well with respect to patient-centeredness. Structuring the work on a ward and fulfilling the physician’s role in an interprofessional team are the most important task for a young resident which require management competencies to enhance trust in the resident by the supervising attending. It seems to be important in the development of undergraduate medical curricula that medical students learn about the perspective of attendings on a residents’ work. For curricular planning, the supervisors’ perspective might be very important to focus more on teamwork and management when designing courses especially for students nearing the transition to residency training. More opportunities to aquire competencies necessary for patient management need to be included in undergraduate medical education to prepare students for the requirements of residency and their personal responsibility as residents.

## Abbreviations

ANOVA: Analysis of variance
EPA: Entrustable professional activity
UKE: University Medical Center Hamburg-Eppendorf

## References

[1] Frank JR, Snell L, Sherbino J, editors. CanMEDS 2015 Physician Competency Framework. Ottawa: Royal College of Physicians and Surgeons of Canada; 2015.

[2] Borleffs JC, ten Cate TJ (2004). Competency-based training for internal medicine. Neth J Med.

[3] Kadmon M, Bender MJ, Adili F, Abab D, Heinemann MK, Hofmann HS (2013). Competency-based medical education: National Catalogue of learning objectives in surgery. Chirurg.

[4] Harris P, Snell L, Talbot M, Harden RM (2010). Competency-based medical education: implications for undergarduate programs. Med Teach..

[5] Albanese MA, Mejicano G, Anderson WM, Gruppen L (2010). Building a competency-based curriculum: the agony and the ecstasy. Av Health Sci Educ theory Pract.

[6] Fischer MR, Bauer D, Mohn K (2015). NKLM-Projektgruppe. Finally finished! National Competence Based Catalogues of learning objectives for undergraduate medical education (NKLM) and dental education (NKLZ) ready for trial. GMS Z Med Ausbild.

[7] Chen HC, van den Broek WE, ten Cate O (2015). The case for use of entrustable professional activities in undergraduate medical eduation. Acad Med.

[8] Ten Cate O (2005). Entrustability of professional activities and competency-based training. Med Educ.

[9] Ten Cate O, Snell L, Carraccio C (2010). Medical competence: the interplay between individual ability and the health care environment. Med Teach.

[10] Wijnen-Meijer M, ten Cate O, van der Schaaf M, Harendza S (2013). Graduates from vertically integrated curricula. Clin Teach.

[11] Rademakers J, De Rooy NC, Ten Cate O (2007). Senior medical students' appraisal of CanMEDS competencies. Med Edu.

[12] Sterkenburg A, Barach P, Kalkman C, Gielen M, ten Cate O (2010). When do supervising physicians decide to entrust residents with unsupervised tasks?. Acad Med.

[13] Kilminster S, Cottrell D, Grant J, Jolly B (2007). AMEE guide no. 27: effective educational and clinical supervision. Med Teach.

[14] Raymond MR, Mee J, King A, Haist SA, Winward ML (2011). What new residents do during their initial months of training. Acad Med.

[15] Touchie C, De Champlain A, Pugh D, Downing S, Bordage G (2014). Supervising incoming first-year residents: faculty expectations versus residents’ experiences. Med Educ.

[16] Wijnen-Meijer M, Van der Schaaf M, Nillesen K, Harendza S, Ten Cate O (2013). Essential facets of competence that enable trust in graduates: a Delphi study among physician educators in the Netherlands. J Grad Med Educ.

[17] Wijnen-Meijer M, van der Schaaf M, Nillesesen K, Harendza S, Ten Cate O (2013). Essential facets of competence that enable trust in medical graduates: a ranking study among physician educators in two countries. Perspect Med Educ.

[18] Brody H, Doukas D (2014). Professionalism: a framework to guide medical education. Med Educ.

[19] Hafferty FW, Castellani B (2010). The increasing complexities of professionalism. Acad Med.

[20] Miles S, Kellett J, Leinster SJ (2017). Medical graduates’ preparedness to practice: a comparison of undergraduate medical school training. BMC Med Educ.

[21] DeLeon S, Mothner B, Middleman A. Improving student documentation using a feedback tool. Clin Teach. 2017; doi: 10.1111/tct.12625. [Epub ahead of print]. 10.1111/tct.12625 28225204

[22] Hauer KE, Durning SJ, Kernan WN, Fagan MJ, Mintz M, O’Sullivan PS, Battistone M, DeFer T, Elnicki M, Harrell H, Reddy S, Boscardin CK, Schwartz M (2008). Factors associated with medical students’ career choices regarding internal medicine. JAMA.

[23] Myers CG, Pronovost PJ (2017). Making management skills a core component of medical education. Acad Med.

[24] Englander R, Flynn T, Call S, Carraccio C, Cleary L, Fulton TB, Garrity MJ, Lieberman SA, Lindeman B, Lypson LM, Minter RM, Rosenfield J, Thomas J, Wilson MC, Aschenbrener AC (2016). Toward defining the foundation of the MD degree: core entrustable professional activities for entering residency. Acad Med.

[25] Swing SR, International CBME Collaborators (2010). Perspectives on competency-based medical education from the learning sciences. Med Teach.

[26] McKenzie S, Mellis C (2017). Practically prepared? Pre-intern student views following an education package. Adv Med Educ Pract.

[27] Warmington S, McColl G (2017). Medical student stories of participation in patient care-related activities: the construction of relational identity. Adv Health Sci Educ Theory Pract.

[28] Mamede S, van Gog T, van den Berge K, van Saase JL, Schmidt HG (2014). Why do doctors make mistakes? A study of the role of salient distrating clinical features. Acad Med.

[29] Norman GR, Eva KW (2010). Diagnostic error and clinical reasoning. Med Educ.

[30] Reilly JB, Ogdie AR, von Feldt JM, Myers JS (2013). Teaching about how doctors think: a longitudinal curriculum in cgonitive bias and diagnostic error for residents. BMJ Qal Saf.

